# A comparative study to evaluate the effect of honey dressing and silver sulfadiazene dressing on wound healing in burn patients

**DOI:** 10.4103/0970-0358.59276

**Published:** 2009

**Authors:** P. S. Baghel, S. Shukla, R. K. Mathur, R. Randa

**Affiliations:** Department of Surgery, MGM Medical College and MY Hospital, Indore, India; 1 Department of Paediatrics, MGM Medical College and MY Hospital, Indore, India

**Keywords:** Honey dressing, silver-sulfadiazine dressing, wound healing in burn

## Abstract

To compare the effect of honey dressing and silver-sulfadiazene (SSD) dressing on wound healing in burn patients. Patients (n=78) of both sexes, with age group between 10 and 50 years and with first and second degree of burn of less than 50% of TBSA (Total body surface area) were included in the study, over a period of 2 years (2006-08). After stabilization, patients were randomly attributed into two groups: ‘honey group’ and ‘SSD group’. Time elapsed since burn was recorded. After washing with normal saline, undiluted pure honey was applied over the wounds of patients in the honey group (n=37) and SSD cream over the wounds of patients in SSD group (n=41), everyday. Wound was dressed with sterile gauze, cotton pads and bandaged. Status of the wound was assessed every third and seventh day and on the day of completion of study. Patients were followed up every fortnight till epithelialization. The bacteriological examination of the wound was done every seventh day. The mean age for case (honey group) and control (SSD group) was 34.5 years and 28.5 years, respectively. Wound swab culture was positive in 29 out of 36 patients who came within 8 hours of burn and in all patients who came after 24 hours. The average duration of healing in patients treated with honey and SSD dressing at any time of admission was 18.16 and 32.68 days, respectively. Wound of all those patients (100%) who reported within 1 hour became sterile with honey dressing in less than 7 days while none with SSD. All of the wounds became sterile in less than 21 days with honey, while tthis was so in only 36.5% with SSD treated wounds. The honey group included 33 patients reported within 24 hour of injury, and 26 out of them had complete outcome at 2 months of follow-up, while numbers for the SSD group were 32 and 12. Complete outcome for any admission point of time after 2 months was noted in 81% and 37% of patients in the honey group and the SSD group. Honey dressing improves wound healing, makes the wound sterile in lesser time, has a better outcome in terms of prevention of hypertrophic scarring and post-burn contractures, and decreases the need of debridement irrespective of time of admission, when compared to SSD dressing.

## INTRODUCTION

Burn management in developing countries is riddled with difficulties. The exact number of cases is difficult to determine; however, in a country like India, with a population of over 1 billion, we would estimate 700,000-800,000 burn admissions annually.[1] Burn injury to the integument causes cellular death, capillary damage in varying degrees and coagulation of proteins. The loss of protective function of the skin as a barrier to micro-organisms results in infection. Immediately after burns the wound is sterile, but within a very short time bacteria contaminating the wound surface begin to multiply and proliferate in the area of the burn wound leading to extensive bacterial colonization. Thus, wounds become a major problem following burns. These patients face a higher morbidity than mortality because of a large uncovered burn surface getting infected, necessitating long periods of dressings, leading to deformities and contractures.[2] Unfortunately, management of the burn wound still remains a matter of debate and an ideal dressing for burn wounds has not been discovered.[2]

Since ancient times, various dressing materials have been used for dressing the burn wounds amniotic membrane, boiled potato peel, banana leaf, soframycin cream, silver sulfadiazene (SSD), skin grafting, epidermal growth factor, honey dressing, etc. Honey, being economical and easily available, makes it a reasonably ideal dressing material in developing countries like India. The present study is aimed to compare the effect of honey dressing and SSD dressing on wound healing.

## MATERIAL AND METHODS

Our study plan was approved by the ethical committee of our institution. Patients (n=78) of both sexes admitted in the burn unit of M.Y. Hospital, Indore, over a period of 2 years (June 2006 to June 2008) with age group between 10 and 50 years and with first and second degree of burn having burn area less than 50% of TBSA total body surface area) were included in a randomized comparative study. Patients on chemotherapy, renal and/or liver failure, immunocompromised state and those with bronchial asthma were excluded. After taking consent from the patients/parents or guardians, patients were randomly attributed into two study groups; Honey group and SSD group, and following data were recorded:

1. Registration data: age, sex, residence, level of education, occupation, marital status.
2. Time of admission: time elapsed since burn and taken to reach the hospital.
3. Investigations: CBC, RBS blood urea, serum creatinine, serum electrolyte.
4. Clinical assessment of the wound: site, affected body surface area, degree, depth, presence or absence of slough, culture sensitivity every seventh day, any additional treatment, outcome.
5. Chronological data: dates of admission and discharge.

Patients were stabilized by supportive treatment, and empirical intravenous antibiotic therapy including ampicillin, gentamicin and metronidazole were started in all patients. Wound swab cultures from three different sites from all patients were taken, at the time of admission and then at every seventh day. Antibiotics were initiated according to the results of bacteriological examination. Intravenous antibiotics were given for minimum 10 days to all the patients with second-degree burns and for 5 days to all the patients with first-degree burns. Wounds were examined carefully and washed with normal saline. Patients included in the honey group were dressed with pure undiluted honey and those in the SSD group with SSD cream, everyday. After application of the dressing material, sterile gauze and cotton pads were applied and wounds bandaged. The status of the wounds was assessed every third and seventh day, and on the day of discharge. Patients were followed up every fortnight for 2 months.

Wound was assessed at third and seventh day and at the time of completion of study. Final outcome was measured after 2 months of follow-up, in terms of complete and incomplete recovery. Complete recovery included complete healing without scar or contracture. Formation of soft scar, hypertrophic scar and/or contracture was taken as incomplete recovery.

## RESULTS

A majority of patients (n=36) reported within 1-8 hours of burn. Out of 78 patients, 54 patients came to the hospital within first 24 hours of burn, while 13 patients came after 24 hours of burn [Table 1]. Other patient characteristics are shown in Table 1. There were no significant differences among these in both groups except that more number of patients in the honey group than in the SSD group presented earlier.

**Table 1:** Characteristics of the patients in the honey and SSD groups

*Patient characteristics*		*Honey group*		*SSD group*		*P-value*
				
		*No. of Cases*	*%*	*No. of Cases*	*%*
Sex	Male	21	57	23	56	0.81*
	Female	16	43	17	44	
	Total	37	100	41	100	
	1st degree	21	57	21	51	0.35*
Degree of burn	2nd degree	16	43	20	49	
	Total	37	100	41	100	
Percentage of burn	<10%	0	0	02	4.8	0.67**
	11-20%	7	18.9	12	29.2	
	21-30%	13	35.1	10	24.3	
	31-40%	08	21.6	06	14.6	
	41-50%	09	24.3	11	26.8	
	Total	37	100	41	100	
	<1 h	05	13	6	15	0.05**
Time of admission	1-8 h	24	65	12	29	
	9-24 h	04	11	14	34	
	25-48 h	00	0	02	5	
	> 48 h	04	11	7	17	
	Total	37	100	41	100	

* Mann-Whitney test

** Pearson chi-square test

100% of patients in the honey group, who presented in less than 1 hour of burn, had their wound swab cultures negative at the time of admission; while corresponding figure for the SSD group was 66%. Patients who came within 1-8 hours following burn, 83% and 75% in two groups, respectively, had wound swab culture positive at the time of admission. All patients in both groups reporting after 24 hours had wound swab culture positive on admission [Table 2].

**Table 2:** Effect of time of reporting after burn on ‘Wound swab culture’ at admission in both groups

*S. No.*	*Time of reporting*	*Honey group*			*SSD group*			*P-value*
			
		*No. of cases*	*+ve*	*−ve*	*No. of cases*	*+ve*	*−ve*	
1	<1 h	05	00	05 (100)	06	2 (34)	4 (66)	0.09
2	1-8 h	24	20 (83)	04 (17)	12	9 (75)	03 (25)	
3	9-24 h	04	02 (50)	02 (50)	14	14 (100)	0	
4	24-48 h	00	-	-	02	02 (100)	0	
5	> 48 h	04	04 (100)	00	07	07 (100)	0	
Total		37	26	11	41	34	7	

Figures in parentheses are in percentage

The average duration of wound healing in patients in the honey group coming within 1 hour, 2-8 hours, 9-24 hours and more than 48 hours was 18.8, 17.8, 21.25 and 14.25 days, respectively. Among patients in the SSD group, average duration of healing was 27.6, 32.4, 32.5, 32.5 and 38.6 days for similar times of reporting [Table 3]. Thus average duration of healing of patients in the honey group was significantly lower than that of patients in the SSD group.

**Table 3:** Effect of time of reporting after burn on ‘Healing’ with treatment in both groups

*S. No.*	*Time to report*	*Honey group*				*SSD group*				*P-value*
				
		*Duration of healing (days)*				*Duration of healing (days)*				
				
		*No. of cases*	*Min*	*Max*	*Avg*	*No. of cases*	*Min*	*Max*	*Avg*	
1	<1 h	05	13	28	18.8	06	22	37	27.6	0.05
2	1-8 h	24	10	31	17.8	12	17	41	32.4	
3	9-24 h	04	17	30	21.2	14	27	40	32.5	
4	25-48 h	00	-	-	-	02	28	37	32.5	
5	> 48 h	04	12	25	14.2	07	28	42	38.5	
Total		37			18.1	41			32.6	

Among patients treated with honey dressing, wound swab culture became negative in less than 7 days, in 62.5%, 50% and 50% of total number of patients reporting in 2-8 hours, 9-24 hours and after 48 hours, respectively [Table 4]. Among patients treated with SSD dressing, none of the patients' wounds became sterile in less than 7 days. Wounds of half (50%) of the patients who presented within an hour of burn became sterile in less than 21 days, those of 33.3% in less than 14 days and of 16.6% in more than 28 days. Patients who presented between 2 and 8 hours (n- 5) 41.6% had their wound sterile in less than 28 days, 33.3% in less than 21 days, 16.6% in more than 28 days and 8.3% in less than 14 days. Patients who presented after 48 hours, 71.4% of these patients had their wound sterile in more than 28 days, 14.2% each in less than 28 and 21 days [Table 4].

**Table 4:** Effect of Honey and SSD to achieve sterilization of wound at different times of reporting after burn

*Time to report*	*Group*	*No. of cases*	*Time taken to sterilize the wound*					*P-value*
				
			*<7 days*	*<14 days*	*<21 days*	*<28 days*	*>28 days*	
<1 h	SSD	06	-	02 (33.3)	03 (50)	-	01 (16.6)	0.01
	Honey	05	05 (100)	-	-	-	-	
1-8 h	SSD	12	-	01 (8.3)	04 (33.3)	05 (41.6)	02 (16.6)	0.03
	Honey	24	15 (62.5)	09 (37.5)	-	-	-	
9-24 h	SSD	14	-	-	06 (42.8)	07 (50)	01 (7.1)	0.04
	Honey	04	02 (50)	01 (25)	01 (25)	-	-	
25-48 h	SSD	02	-	-	01 (50)	01 (50)	-	-
	Honey	00	-	-	-	-	-	
> 48 h	SSD	07	-	-	01 (14.2)	01 (14.2)	05 (71.4)	0.02
	Honey	04	02 (50)	-	02 (50)	-	-	

Figures in parentheses are in percentage

Among 33 patients treated with honey dressing who reported within 24 hours, 26 patients had complete recovery while 7 had incomplete. Out of a total 37 patient treated with honey dressing, 30 (81%) had complete recovery. In the SSD group, out of 32 patients presenting within 24 hours, only 12 patients had complete recovery and out of a total of 41 patients, only 15 (37%) achieved complete recovery. These differences were statistically significant [Table 5].

**Table 5:** Final outcome in the ‘honey group’ and the ‘SSD group’ at different times of reporting after burn

*Time to report*	*Honey group*			*SSD group*			*P-value*
		
	*No. of cases*	*Complete*	*Incomplete*	*No. of cases*	*Complete*	*Incomplete*	
<1 h	05	02	03	06	03	03	0.02
1-8 h	24	21	03	12	04	08	
9-24 h	04	03	01	14	05	09	
25-48 h	00	-	-	02	01	01	
> 48 h	04	04	-	07	02	05	
Total	37	30 (81%)	07 (19%)	41	15 (37%)	26 (63%)	

## DISCUSSION

For at least 2700 years, honey has been used to treat a variety of ailments through topical application, but only recently, its antiseptic and antibacterial properties have been chemically explained. Although, in 2001, in their excellent review of randomized controlled trials comparing honey with other materials, Moore *et al*,[3] concluded that the confidence in honey as a useful treatment option for superficial wounds or burns is low and there is a definite biological plausibility for the same. But recently in 2004, Professor Peter Molan from New Zealand, based on his work at Honey Research Unit at the University of Waikato, stated that a particular type of honey might be useful in treating methicillin-resistant *Staphylococcus aureus* (MRSA) infections.[4]

Antibacterial properties of honey result from its low water activity which causes osmosis, its hydrogen peroxide content[5] and its high acidity.[6] Being primarily a saturated mixture of two monosaccharides, this mixture has a low water activity. Since most water molecules get associated with the sugars, only a few remain for micro-organisms, rendering a poor environment for their growth. Hydrogen peroxide in honey is activated by dilution; however, unlike medical hydrogen peroxide, commonly 3% by volume, it is present in a concentration of only 1 mmol/L in honey. Iron in honey oxidizes the oxygen free radical released by the hydrogen peroxide. When used topically (as for example, a wound dressing), hydrogen peroxide is produced by dilution with body fluids. As a result, hydrogen peroxide is released slowly and acts as an antiseptic. The pH of honey is commonly between 3.2 and 4.5. This relatively acidic pH level prevents the growth of many bacteria.[6] The antibacterial activity of honey is mainly due to inhibins in honey. These inhibins consist of hydrogen peroxide, flavinoids, and phenolic acids, plus many other unidentified substances.[7,8] Some studies suggest that the topical use of honey may reduce odours, swelling and scarring when used to treat wounds; it may also prevent the dressing from sticking to the healing wound.[6]

Burn patients have a higher morbidity than mortality because burn wound, due to the presence of necrotic tissue, has great chances of infection and thus requires long periods of dressings, leading to deformities and contracture.[2] Delayed reporting has been found to be an important factor that causes an increase in wound infection and thus morbidity.[9] This is a major problem in the third world countries like India, owing to poor transport condition, illiteracy and relative inaccessibility of tertiary health-care centres. Delay and inadequate fluid resuscitation and overwhelming infection were the major factors in the morbidity and mortality.[10]

In patients with severe burns, wound infection and contamination frequencies have been found to be higher for all admission time points.[9] Infection is one of the most frequent complications of wound healing despite the use of antibiotics and a modern sterile technique; it accounts for considerable patient morbidity, discomfort and prolonged hospitalization and it must be avoided to permit proper healing.[11]

Honey dressing decreases the average duration of healing as compared to the SSD dressing. The healing process requires clearance of pathogenic organisms. Since antibiotics are ineffective in this situation and antiseptics cause tissue damage, the healing process is slow.[12] Honey is reported to cause no tissue damage and appears to actually promote the healing process. There are also numerous reports of sugar being used as a wound dressing.[13–18]

The results show that the average duration of healing was increased, as there was delay in admission in hospital, but the increase in duration of healing was more with the SSD dressing than with honey dressing. Honey therapy was seen to decrease the levels of serum lipid peroxide; while there was a mild increment in serum ceruloplasmin levels, there was no significant effect on serum uric acid levels as compared to SSD treatment. Honey therapy seems to accelerate the process of healing. It has a more positive effect on reducing the oxidative stressful state in burn trauma when compared to SSD treatment, resulting in results in rapid wound healing.[19] Patients who reached the hospital before 24 hours of burn had an average duration of healing of 19.28 and 30.83 days for honey and SSD groups. Those who reported after 48 hours had average duration of healing 14.25 and 38.57 days with honey and SSD dressing, respectively [Table 3]. Similar results were found in the previous study done by Subrahmanyam,[20] where 84% and 72% showed satisfactory epithelialization by the seventh day with honey and SSD dressing, respectively. Epithelialization occurred in 100% and 84% of the patients by the 21st day in wounds treated with honey and SSD dressing, respectively. Histological evidence of reparative activity reached 100% by 21 days with the honey dressing and 84% with SSD.[20]

In our study, 100% of wounds of patients who reported within 1 hour for admission and were treated with honey dressing became sterile in less than 7 days. 62.5%, 50%, 50% of the patients treated with honey dressing who reported within 2- 8 hours, 9-24 hours and more than 48 hours, respectively, attained wound sterility in less than 7 days [Table 4]. This observation is comparable with another study of Subrahmanyam.[21] In the 52 patients treated with honey, 91% of wounds were rendered sterile within 7 days.[21] 37.5% of patients' wound became sterile in less than 14 days who reported within 2-8 hours [Table 4]. Antibacterial activity is attributed by several authors to the high osmolarity of the sugar or honey.[14,22–24] Of the wounds treated with honey, 87% healed within 15 days as against 10% in the control group.[21]

SSD dressing did not have any added benefit over honey dressing in terms of healing and making wound sterile. It is concluded that although there is evidence of antibacterial effect, there is no direct evidence of improved healing or reduced infection by SSD dressing.[25] When both type of dressings were compared, early subsidence of acute inflammatory changes, better control of infection and quicker wound healing were observed with honey dressing, while in the SSD-treated wounds sustained inflammatory reaction was noted even on epithelialization.[20]

Also, it was clear that increase in time of admission had adverse effect on wound healing and complication at follow-up. This is more so for SSD dressing. Subramanyam,[21] also concluded that relief of pain, lower incidence of hypertrophic scar and postburn contracture, low cost and easy availability make honey an ideal dressing in the treatment of burns.

## CONCLUSION

Delay in hospital admission increases wound contamination and infection thereby delaying wound healing which has a detrimental effect on final outcomes. Since honey dressing improves wound healing by rendering it sterile in lesser duration of time, wounds thus treated have a better outcome in terms of hypertrophic scarring and post-burn contractures; this is due to the fact that early healing mitigates the need for debridement at when compared to SSD dressing. Hence, Honey dressing is a better option for dressing in burns, in terms of decreased morbidity, economy, patient well-being and speedy rehabilitation.
